# Effects of Stocking Density and Body Size on Oxygen Consumption and Ammonia Excretion in Silverside (*Odontesthes bonariensis*) Reared in a Recirculating Aquaculture System

**DOI:** 10.3390/ani16142114

**Published:** 2026-07-08

**Authors:** Carlos Andres Mendez, Carla Galleguillos, Cristian C. Harris-Toro, María Luisa Nava, María Cristina Morales

**Affiliations:** 1 Departamento de Acuicultura, Facultad de Ciencias del Mar, Universidad Católica del Norte, Larrondo 1281, Coquimbo 1780000, Chile; carla.galleguillos@ucn.cl (C.G.); cristian.harris@ucn.cl (C.C.H.-T.); mlnava@ucn.cl (M.L.N.); 2 Núcleo FIGEMA-Tec., Universidad Católica del Norte, Larrondo 1281, Coquimbo 1780000, Chile

**Keywords:** *Odontesthes bonariensis*, recirculating aquaculture system (RAS), stocking density, body size, specific dynamic action, bioengineering parameters

## Abstract

Optimizing oxygen supply and nitrogen removal in recirculating aquaculture systems (RAS) requires species-specific metabolic benchmarks. This study quantified the effects of body size and stocking density on oxygen consumption and total ammonia nitrogen (TAN) excretion in the silverside *Odontesthes bonariensis* under controlled RAS conditions. A 2 × 2 factorial design was used to compare small (48–140 g) and large (>140–250 g) fish stocked at low (3.2 kg m^−3^) and high (6.2 kg m^−3^) densities. Oxygen consumption was significantly influenced by both factors, with mean routine rates ranging from 146.12 ± 35.07 mg O_2_ kg^−1^ h^−1^ in high-density large fish to 226.31 ± 50.71 mg O_2_ kg^−1^ h^−1^ in low-density small fish. Smaller fish exhibited higher mass-specific rates, and individuals held at lower densities consumed more oxygen, consistent with allometric scaling and density-dependent metabolic suppression. In contrast, TAN excretion was unaffected by size or density, with mean values ranging from 4.59 ± 0.77 to 10.81 ± 3.76 mg TAN kg^−1^ h^−1^ across treatments, indicating stable protein catabolism under a uniform feeding regime. Both parameters displayed pronounced diurnal fluctuations, with postprandial peaks associated with specific dynamic action: oxygen consumption fluctuated between 61.75 and 333.03 mg O_2_ kg^−1^ h^−1^, while TAN excretion ranged from 0 to 78.63 mg TAN kg^−1^ h^−1^ over 24 h cycles. These findings demonstrate that oxygen demand in *O. bonariensis* is strongly modulated by bioenergetic scaling and stocking density (ranging from 146 to 226 mg O_2_ kg^−1^ h^−1^), whereas ammonia excretion (4.6–10.8 mg TAN kg^−1^ h^−1^) is primarily driven by dietary input. These results provide species-specific baseline benchmarks for aeration sizing and biofilter design, thereby supporting the sustainable intensification of silverside aquaculture in RAS.

## Simple Summary

This study measured how fish size and how tightly fish are kept affect oxygen use and ammonia waste in a recirculating aquaculture system, using the silverside (*Odontesthes bonariensis*) as a model. We tested small and large fish at two stocking densities and monitored oxygen consumption and ammonia release over daily cycles while feeding them the same diet. We found that smaller fish and fish kept at lower densities used more oxygen per kilogram of body weight, while ammonia production per kilogram stayed similar across sizes and densities and depended mainly on food intake. Both oxygen use and ammonia release rose after feeding and varied throughout the day. These results provide practical numbers that fish farmers and system designers can use to size aeration and filtration equipment more accurately, helping to keep water quality safe, reduce energy and treatment costs, and support more sustainable and productive silverside farming.

## 1. Introduction

The silverside, *Odontesthes bonariensis* (family Atherinidae), is native to shallow lakes and lagoons of Buenos Aires Province, Argentina [1]. This species holds high socio-economic value for both commercial and recreational fisheries and is considered a promising candidate for aquaculture diversification [2]. Its market demand and commercial viability further support its cultivation potential [3]. In Argentina, pejerrey represents the second most economically important inland fishery resource, with production destined for domestic consumption and international markets, including Bolivia, Ukraine, Lithuania, Russia, and the United States [4,5]. Due to its favorable biological and ecological traits, *O. bonariensis* has been introduced into multiple countries worldwide, including Brazil, Bolivia, Chile, Peru, Paraguay, Uruguay, Italy, France, Morocco, Japan, and Israel [6,7,8]. In Chile, the species was introduced in 1940 for recreational fishing in temperate lakes, reservoirs, and artificial ponds [9].

Aquaculture development for this species has primarily focused on larval and juvenile production for restocking programs supported by provincial governments in Argentina [10,11]. However, the expansion of intensive production systems has been constrained by economic, social, and biological factors, as well as by gaps in technical and scientific knowledge [12]. Among the biological limitations, relatively slow growth rates compared to other commercial fish species have reduced productivity [10,11,13]. Nevertheless, suboptimal culture conditions may partly account for this limitation, as semi-intensive approaches have demonstrated improved growth compared to traditional intensive methods, indicating that the species’ full growth potential has not yet been fully exploited [14]. Furthermore, the absence of structured genetic improvement programs targeting enhanced growth and disease resistance continues to hinder the advancement of efficient aquaculture practices [15]. In *O. bonariensis* culture, limited information on optimal environmental and nutritional conditions across developmental stages contributes to reproductive failures, feed inefficiencies, and water quality deterioration [16,17]. These constraints ultimately impede the sustainable expansion of silverside aquaculture and are partly attributable to an incomplete understanding of the species’ bioenergetics.

A fundamental challenge in aquaculture system design is determining the maximum stocking density that can be maintained within a culture unit, which depends on water quality requirements and species-specific bioengineering parameters [18]. Among the most relevant bioengineering parameters are oxygen consumption rate and ammonia excretion rate, as these are critical for water quality management and serve as direct indicators of fish metabolic activity [19,20,21]. With the continued expansion of aquaculture, water quality—particularly the concentration of un-ionized ammonia—has become a global environmental concern [22]. Ammonia is the primary end product of protein metabolism in fish and is excreted mainly across the gills. Its un-ionized form (NH_3_) is highly toxic and can adversely affect growth performance, immune function, and survival of cultured organisms [23,24]. The proportion of un-ionized ammonia increases with rising temperature, pH, and salinity [25,26]. In recirculating aquaculture systems (RASs), ammonia accumulation is typically controlled through nitrification and denitrification processes carried out by specialized microorganisms, which sequentially convert ammonia to nitrite, nitrate, and ultimately to gaseous nitrogen [27].

Ammonia excretion rates are influenced by both endogenous and exogenous factors, including diet composition, stocking density, and environmental conditions [28,29]. Stocking density is particularly critical, as it directly affects water quality, feed efficiency, growth performance, and overall fish welfare [30]. High-protein diets and elevated stocking densities increase ammonia loading within the system, necessitating continuous monitoring to prevent toxic effects [31].

Despite the economic importance and recognized aquaculture potential of *O. bonariensis*, no published data exist on its oxygen consumption rate or ammonia excretion rate under any intensive or semi-intensive culture system. Furthermore, bioengineering metabolic parameters are absent for all members of the family Atherinidae under RAS conditions. This knowledge gap limits the accurate estimation of carrying capacity, oxygen supply requirements, and biofilter dimensioning for silverside culture. The combined effects of stocking density and body size on metabolic demand and nitrogen excretion in this species have therefore not been previously characterized.

Therefore, the aim of this study was to evaluate the influence of stocking density and body size on oxygen consumption and ammonia excretion rates of *O. bonariensis* cultured in a recirculating aquaculture system in order to generate species-specific metabolic parameters that support system optimization and sustainable production strategies.

## 2. Materials and Methods

### 2.1. Experimental Animals

Fish were captured using gill nets in the Hurtado River and transported to the Central Marine Aquaculture Laboratory, Faculty of Marine Sciences, Universidad Católica del Norte, Coquimbo, Chile (29°57′ S–71°21′ W). The fish were then transferred from cultivation ponds to circular cement tanks (12,000 L) supplied with a constant water flow (5 L s^−1^) and continuous aeration for acclimation to the experimental conditions. Fish were acclimated to experimental conditions for 125 days prior to the start of measurements. During the acclimation period, fish were fed daily at 1% body weight, and water quality was maintained as described. Water quality parameters were maintained as follows: temperature 16.51 ± 0.06 °C, dissolved oxygen 9.72 ± 0.07 mg L^−1^, and pH 8.96 ± 0.09. A total of 189 fish were selected and distributed into four experimental groups defined by the combination of two stocking densities and two size ranges: low density–small size (D1S1), low density–large size (D1S2), high density–small size (D2S1), and high density–large size (D2S2). The number of fish per tank for each treatment was as follows: D1S1 = 13 fish tank^−1^, D1S2 = 10 fish tank^−1^, D2S1 = 22 fish tank^−1^, and D2S2 = 18 fish tank^−1^. Stocking densities were 3.2 kg m^−3^ for the low-density condition (D1) and 6.2 kg m^−3^ for the high-density condition (D2), while size ranges were 48–140 g for S1 and >140–250 g for S2. Each combination was carried out in triplicate, with fish randomly distributed among the culture tanks. These densities were selected to represent conditions consistent with semi-intensive silverside culture in Chile and Argentina, where operational stocking densities typically range between 2 and 8 kg m^−3^ for this species. Since no prior data existed for *O. bonariensis* in RAS, conservative levels were chosen to generate baseline bioengineering parameters while ensuring animal welfare and stable water quality.

### 2.2. Experimental Design

To determine the ammonia excretion rate, a recirculating aquaculture system (RAS) with a total volume of 7 m^3^ was used. The system consisted of twelve cylindrical–conical tanks (each 500 L) with dimensions of 1 m in diameter and 1 m water column height. The tanks were connected to a settling tank (1000 L) and a sand filter (10.8 m^3^ h^−1^; Emaux, model P500, Emaux Water Technology Co., Ltd., Zhongshan, China). The system also included a 200 L submerged moving-bed biofilter filled with Kaldnes media (AnoxKaldnes, Lund, Sweden) (specific surface area: 600 m^2^ m^−3^) and a UV sterilization unit (20 W; BioLight, model WUV20-1, Bio Light SpA, Santiago, Chile). All components were connected by a centrifugal pump (1 HP; ESPA, model SILEN 100, ESPA S.A., Girona, Spain) with a maximum flow rate of 14 m^3^ h^−1^. Each rearing tank was equipped with a centrally positioned Aero-Tube™ air diffuser hose (Colorite Aero-Tube, Ridgefield, CT, USA) and a hydraulic aeration pipe connected to a 2.5 HP blower (Sweetwater^®^, Pentair, Golden Valley, MN, USA). A water exchange rate of 0.77 m^3^ h^−1^ was maintained, resulting in a hydraulic retention time of 1.7 h per tank. A natural photoperiod and rearing conditions similar to those used in culture systems were maintained. The experimental period lasted seven days, during which metabolic measurements of oxygen consumption and TAN excretion were conducted. These measurements were performed simultaneously across all three replicate tanks assigned to each treatment. Each 24 h measurement cycle was repeated on three consecutive occasions per tank to obtain stable estimates of metabolic rates. The mean value across the three measurement cycles was calculated for each tank, and the tank mean was used as the independent experimental unit for statistical analysis, yielding n = 3 independent observations per treatment.

### 2.3. Determination of Oxygen Consumption

The oxygen consumption rate (OCR) was determined through a mass balance approach under non-steady-state conditions, considering each culture unit as an open respirometer exposed to the atmosphere under standard culture conditions [32,33]. At the start of each 6 h measurement block, the air supply was switched off, and all water inlet and outlet valves were closed, isolating each culture tank as a sealed respirometer. Dissolved oxygen (DO) concentration was recorded at 1 h intervals using a calibrated multiparameter probe (HQ40d, Hach Company, Loveland, CO, USA). Measurements within each block were terminated when dissolved oxygen reached 70% of air saturation (≥6.90 mg O_2_ L^−1^ at the experimental temperature and local atmospheric pressure of Coquimbo, Chile), ensuring that recorded values reflected aerobic metabolic rates under non-critically limiting oxygen conditions and remained well above the critical oxygen tension (Pcrit) associated with acute metabolic suppression in freshwater teleosts (~3.5–4.5 mg O_2_ L^−1^). Between measurement blocks, aeration and water circulation were restored until dissolved oxygen returned to initial saturation levels before the next block commenced. Since the system was completely sealed during measurements, with no water exchange or aeration operating, no correction for oxygen reaeration or atmospheric diffusion was required. This temporal resolution differs from that used for TAN measurements (Section 2.4), where water samples were collected every 2 h within 8 h batch blocks due to analytical constraints of the colorimetric method. The hourly specific oxygen consumption rate was calculated using the following equation:

(1) $$\mathrm{Oxygen}\;\mathrm{consumption}\;\mathrm{rate}\;(\mathrm{mg}\;O_{2}\;{\mathrm{kg}}^{-1}\;\mathrm{fish}\;h^{-1})\;=\;\left.\left(\frac{Ct1-Ct0}{t1-t0}\right.V\right)/B$$

where Ct_1_ is the oxygen concentration at time t_1_; Ct_0_ is the concentration at time t_0_; V is the tank volume in liters (L); and B is the total fish biomass (kg).

Since the system was completely sealed during measurements, with no water exchange or aeration, no correction for oxygen reaeration or atmospheric diffusion was required. The mean OCR for each treatment was calculated as the average of all valid 1 h intervals across the three replicate measurement cycles.

### 2.4. Determination of Ammonia Excretion

Measurements were conducted in three 8 h blocks (08:00–16:00, 16:00–00:00, and 00:00–08:00) in triplicate under closed-flow conditions in each rearing tank to determine the diurnal cycle of ammonia excretion rate across the four treatments. The ammonia excretion rate was determined using a mass balance approach under closed-flow (batch) conditions [34]. During each batch period, the connection between the culture tanks and the biofilter chamber was closed using a manual isolation valve, thereby ensuring that nitrification did not remove TAN from the water during excretion measurements. Aeration in the systems was maintained at the normal flow rate to preserve homogeneous conditions throughout the entire batch period. The maximum biofilter disconnection period was 8 h per batch block. During this period, dissolved oxygen remained above 9.5 mg L^−1^ and TAN accumulation did not exceed 0.61 mg L^−1^, resulting in un-ionized ammonia (NH_3_) levels below 0.03 mg L^−1^, well below the chronic toxicity threshold of 0.05–0.1 mg L^−1^ for freshwater teleosts [23]. These conditions are consistent with published protocols for batch TAN measurement in RAS [34] and indicate that the brief biofilter disconnection is unlikely to have induced measurable physiological stress in the fish. Water samples (10 mL) were collected in triplicate from each tank at 0 h and subsequently every 2 h during batch operation. The total ammonia nitrogen (TAN) concentration was quantified colorimetrically using the salicylate method (Hach Method #8155, low-range: 0.01–0.50 mg NH_3_-N L^−1^) with a Hach DR3900 spectrophotometer (Hach Company, Loveland, CO, USA). The instruments were verified using certified reference standards (Hach AccuVac^®^ ampules, Company, Loveland, CO, USA). Each sample was processed in triplicate, and any dataset showing a coefficient of variation (CV) greater than 5% was subjected to reanalysis. Un-ionized ammonia (mg NH_3_-N L^−1^) was calculated from TAN using pH and temperature values according to the formula proposed by Emerson et al. [35]. The specific hourly TAN excretion rate was calculated using the following equation:

(2) $$\mathrm{TAN}\;\mathrm{excretion}\;\mathrm{rate}\;(\mathrm{mg}\;\mathrm{TAN}\;{\mathrm{kg}}^{-1}\;\mathrm{fish}\;h^{-1})\;=\;\;\left.\left(\frac{Ct1-Ct0}{t1-t0}\right.V\right)/B\;\;\;\;\;$$

where Ct_1_ is the TAN concentration at time t_1_; Ct_0_ is the concentration at time t_0_; V is the tank volume in liters (L); and B is the total fish biomass (Kg).

During the experimental period, dissolved oxygen, temperature, and pH were measured using a Hach HQ40d multiparameter probe. Nitrate levels were recorded weekly using Method 8039 (Cadmium Reduction Method, 0–30.0 mg·L^−1^ NO_3_-N), and nitrite was measured using Method 8507 (Diazotization Method, 0–0.300 mg·L^−1^ NO_2_-N) [36]. Total alkalinity was determined by titration using the Bromophenol Blue method with the HI3811 alkalinity test kit (Hanna Instruments, Woonsocket, RI, USA). Total suspended solids (TSS) were measured following Method 2540D [37].

### 2.5. Feeding Regime

The fish were fed a commercial diet (Biomar ORB Intro, BioMar Chile S.A. Puerto Montt, Chile) containing 50.13–54.13% crude protein, 18.5–20% lipids, a maximum of 10% moisture, and up to 12% ash. Feeding was carried out twice daily (08:00–09:00 h and 16:00–17:00 h) at a rate of 1% of body weight, adjusted biweekly according to total biomass. A feeding rate of 1% body weight day^−1^ was selected as it falls within the range documented for adult *O. bonariensis* (0.5–2% BW day^−1^ at 16–18 °C) and represents a moderate, conservative ration that minimizes overfeeding-related water quality deterioration while maintaining fish at routine (non-maximal) metabolic states, which is the target condition for bioengineering parameter estimation [18]. This approach is consistent with protocols used in previous metabolic studies with teleosts of similar body size [34].

### 2.6. Apparent Nitrogen Balance Assessment

To quantify the proportion of ingested nitrogen recovered as TAN excreted, the dietary nitrogen input was calculated for each treatment as

(3) $$\mathrm{Feed}\;N\;\mathrm{input}\;(\mathrm{mg}\;N\;{\mathrm{day}}^{-1})=\left.\left(\frac{feed\;amount\;({g\;day}^{-1})\times crude\;protein\;content\;(\%)}{6.25\times 1000}\right.\right)\;\;$$

where the feed amount was determined as the product of total tank biomass (g) and the feeding rate (1% body weight day^−1^), and 6.25 is the nitrogen-to-protein conversion factor. The percentage of dietary nitrogen recovered as TAN (TAN recovery, %) was then calculated as

(4) $$\mathrm{TAN}\;\mathrm{recovery}\;(\%)=\left.\left(\frac{daily\;TAN\;excreted\;({mg\;TAN\;day}^{-1})}{Feed\;N\;input\left({mg\;N\;day}^{-1}\right)}\right.\right)\times 100$$

where daily TAN excreted per tank was obtained by multiplying the specific daily excretion rate (mg TAN g fish^−1^ day^−1^) by the total tank biomass (g).

It was assumed that the offered ration was completely consumed, as no visible feed residues were observed during the experimental period. This assumption is a recognized limitation of the present study; actual individual feed intake was not quantified, and fecal nitrogen losses and urea excretion were not measured. Therefore, all derived nitrogen balance values should be interpreted as apparent estimates throughout this manuscript.

To quantify the absolute amount of nitrogen excreted as TAN per tank per day, the TAN excreted (mg N tank^−1^ day^−1^) was calculated for each treatment as follows:

(5) $$\mathrm{TAN}\;\mathrm{excreted}\;(\mathrm{mg}\;N\;\tan k^{-1}\;{\mathrm{day}}^{-1})\;=\;\left(\mathrm{TAN}\;\mathrm{rate}(\frac{mg\;N}{Kg\;fish\;h}\right)\times Biomass\;\left(Kg\;tank^{-1}\right)\times 24\;h)$$

### 2.7. Statistical Analysis

All data are presented as mean ± standard error of the mean (S.E.). Each tank was considered an independent experimental unit (n = 3 per treatment). Oxygen consumption and TAN excretion were analyzed using a two-way ANOVA with stocking density and body size as fixed factors, including their interaction term. When significant main effects were detected, Tukey’s HSD post hoc test was applied. Temporal variations were evaluated using a repeated-measures ANOVA, with time (hour) as the within-subject factor. When the assumption of sphericity was violated, Greenhouse–Geisser corrections were applied. Prior to all analyses, normality of residuals (Shapiro–Wilk test) and homogeneity of variances (Levene’s test) were verified. A significance level of α = 0.05 was adopted for all statistical tests. Post hoc statistical power analysis was performed using the pwr package in R to estimate the observed power (1 − β) for each two-way ANOVA main effect, based on Cohen’s f effect sizes derived from partial eta-squared values (f = √[η^2^p/(1 − η^2^p)]), with n = 3 replicates per treatment. Results are reported alongside the F-statistics, degrees of freedom, and exact *p*-values for all main effects and interactions in the Results section. All analyses were conducted using R software (version 4.4.2; R Core Team) [38].

## 3. Results

### 3.1. Water Quality

The average values of the water quality parameters are presented in Table 1. The physicochemical parameters of the water in the culture tanks were similar across all treatments, with no significant differences (*p* > 0.05). The average concentrations of nitrogenous compounds in the RAS were TAN (NH_3_–N), 0.27 ± 0.02 mg L^−1^; ammonia (NH_3_), 0.065 ± 0.005 mg L^−1^; nitrite (NO_2_–N), 0.03 ± 0.02 mg L^−1^; and nitrate (NO_3_–N), 10.75 ± 1.64 mg L^−1^. Alkalinity averaged 193.54 ± 13.31 mg CaCO_3_ L^−1^, and total suspended solids (TSS) were 21.29 ± 5.67 mg L^−1^.

### 3.2. Oxygen Consumption

**Figure 1:**
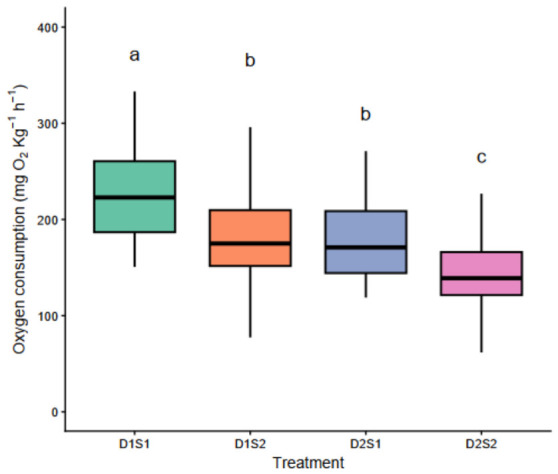
Mass-specific oxygen consumption (mg O_2_ kg^−1^ h^−1^) of *Odontesthes bonariensis* under four experimental treatments combining two stocking densities (D1: low; D2: high) and two body sizes (S1: small; S2: large). Different lowercase letters above boxes indicate significant differences among treatments (*p* < 0.05, ANOVA).

High consistency was observed among replicate tanks within each treatment (coefficient of variation < 15%), indicating adequate experimental reproducibility. Two-way ANOVA revealed significant main effects of both body size (F (1,8) = 7.66, *p* = 0.025, η^2^p = 0.489) and stocking density (F (1,8) = 11.87, *p* = 0.009, η^2^p = 0.597) on oxygen consumption rate. The interaction between body size and stocking density was not significant (F (1,8) = 0.11, *p* = 0.54, η^2^p = 0.014). These results indicate that both factors independently and additively influenced mass-specific oxygen demand in *O. bonariensis*. The mean routine oxygen consumption rates were 226.31 ± 50.71 mg O_2_ kg fish^−1^ h^−1^ for D1S1, 186.32 ± 49.12 mg O_2_ kg fish^−1^ h^−1^ for D1S2, 177.56 ± 37.61 mg O_2_ kg fish^−1^ h^−1^ for D2S1, and 146.12 ± 35.07 mg O_2_ kg fish^−1^ h^−1^ for D2S2. Significant differences were observed among treatments (*p* < 0.05) (Figure 1).

**Figure 2:**
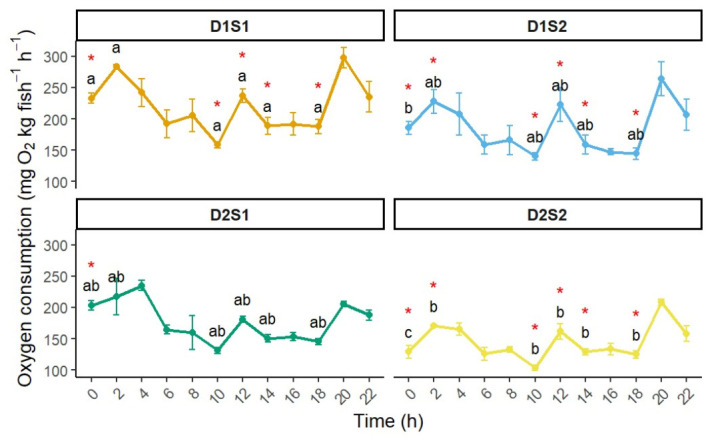
Diurnal variation in mass-specific oxygen consumption (mg O_2_ kg fish^−1^ h^−1^) of *Odontesthes bonariensis* under four experimental treatments. Values are presented as mean ± S.E. at 2 h intervals over a 24 h cycle. Red asterisks indicate time points showing significant differences among treatments at post hoc comparisons (*p* < 0.05, Tukey HSD). Different letters above data points indicate statistically significant differences among means according to Tukey HSD multiple comparisons. The treatment × time interaction was not significant (F (9,24) = 0.82, *p* = 0.60), indicating that treatments did not differ in their diurnal metabolic pattern.

Daily oxygen consumption exhibited a pronounced diurnal pattern, with significant fluctuations throughout the 24 h cycle (Figure 2). These variations were primarily associated with postprandial metabolic activity. Oxygen consumption values ranged from 61.75 to 333.03 mg O_2_ kg fish^−1^ h^−1^. The absence of a significant interaction between treatment and time indicates that, although temporal variation occurred, stocking density and body size did not modify the overall circadian pattern of oxygen consumption. Repeated-measures ANOVA showed a significant effect of time on oxygen consumption (F (3,24) = 12.5, *p* < 0.001), consistent with postprandial metabolic peaks. However, the treatment × time interaction was not significant (F (9,24) = 0.82, *p* = 0.60, η^2^p = 0.24), indicating that stocking density and body size did not modify the diurnal pattern of oxygen consumption.

### 3.3. Ammonia Excretion Rate

**Figure 3:**
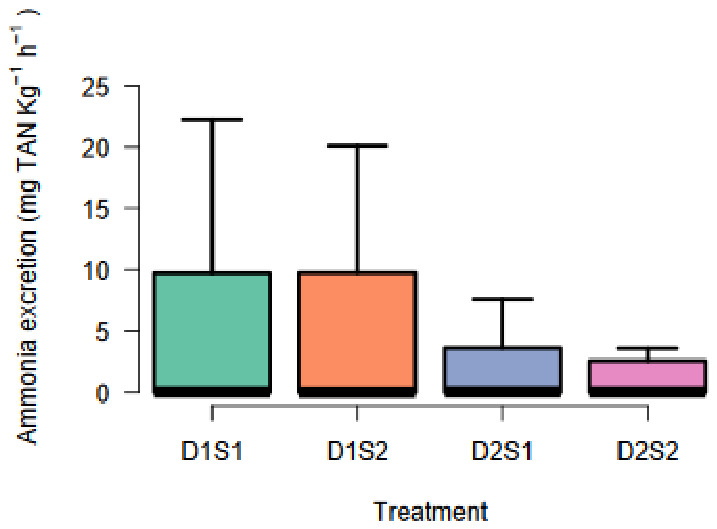
Mass-specific ammonia excretion (mg TAN kg^−1^ h^−1^) of *Odontesthes bonariensis* under four experimental treatments. No significant differences were observed in ammonia excretion rates among treatments (*p* > 0.05).

No mortality was recorded among the cultured fish in any of the treatments. The daily ammonia excretion rate (TAN) under routine conditions was 9.47 ± 3.12 mg TAN kg fish^−1^ h^−1^ for D1S1, 10.81 ± 3.76 mg TAN kg fish^−1^ h^−1^ for D1S2, 5.57 ± 2.00 mg TAN kg fish^−1^ h^−1^ for D2S1, and 4.59 ± 0.77 mg TAN kg fish^−1^ h^−1^ for D2S2. Two-way ANOVA revealed no significant main effects of body size (F (1,8) = 3.93, *p* = 0.083, η^2^p = 0.329) or stocking density (F (1,8) = 0.01, *p* = 0.94, η^2^p = 0.001) on TAN excretion rate, and no significant body size × stocking density interaction was detected (F (1,8) = 0.21, *p* = 0.66). Post hoc power analysis indicated an observed power of 30% for the density effect and 6% for the size effect, suggesting that the non-significant results may partly reflect limited statistical power associated with the low replication (n = 3) and high within-treatment variability in TAN excretion (Figure 3).

**Figure 4:**
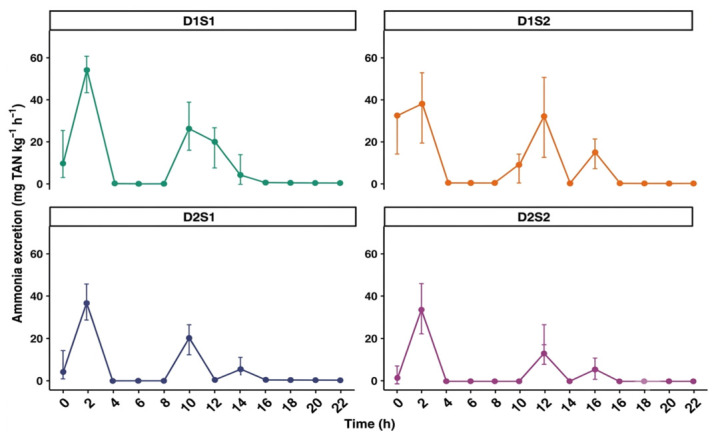
Diurnal variation in TAN excretion (mg TAN kg fish^−1^ h^−1^) of *Odontesthes bonariensis* under four experimental treatments. Values are expressed as mean ± S.E. at 2 h intervals over a 24 h cycle. Peaks in TAN excretion were observed shortly after feeding, followed by a rapid decline toward baseline levels. The treatment × time interaction was not significant (F (6,16) = 0.62, *p* = 0.71), indicating that the temporal pattern of TAN excretion was consistent across all treatments.

A diurnal pattern of TAN excretion was observed in all groups, characterized by three main peaks: at approximately 02:00 h, approximately 2 h after feeding (10:00 h), and at 14:00 h (Figure 4). Repeated-measures ANOVA showed a significant effect of time on TAN excretion (F (2,16) = 4.1, *p* = 0.036), reflecting postprandial peaks. Neither the main effect of treatment (F (3,8) = 1.8, *p* = 0.22) nor the treatment × time interaction (F (6,16) = 0.62, *p* = 0.71, η^2^p = 0.19) was statistically significant. TAN excretion values fluctuated between 0 and 78.63 mg TAN kg^−1^ fish h^−1^ over the 24 h period.

### 3.4. Apparent Nitrogen Balance

Table 2 summarizes the apparent nitrogen balance and derived parameters for each treatment. Dietary nitrogen intake was constant (0.83 mg N g^−1^ day^−1^) due to the fixed ration. The apparent TAN recovery was lower in high-density treatments (13–16%) compared to low-density treatments (27–31%), although these differences were not statistically significant (*p* > 0.05). Total excretion per tank varied between 341.5 ± 57.3 and 415.1 ± 144.4 mg N tank^−1^ day^−1^, reflecting differences in total biomass and specific excretion rates. These differences in apparent TAN recovery between density treatments were not statistically significant (F (1,8) = 4.76, *p* = 0.061, η^2^p = 0.373), partly reflecting the high variability in TAN excretion and the assumed complete feed consumption, which may have introduced uncertainty in the estimated recovery values.

## 4. Discussion

Environmental parameters remained within the optimal ranges previously reported for *O. bonariensis* cultures [12,14,39,40]. In aquaculture systems, dissolved oxygen (DO) concentrations should be maintained above 4 mg L^−1^ to prevent physiological stress and preferably between 5 and 9 mg L^−1^ for species with high metabolic demand [41]. Adequate oxygen availability promotes efficient feed conversion, optimal growth performance, and high survival rates [42,43]. In the present study, DO levels (9.72–9.76 mg L^−1^) were consistently above these thresholds, ensuring that the measured metabolic rates reflected treatment effects rather than environmental stress or oxygen limitation.

During the experimental period, pH values remained within the 7.0–9.0 interval (8.96–8.98), a range documented to support both fish welfare and nitrifying bacterial activity [44]. Although the recorded pH was relatively high for freshwater systems, TAN concentrations remained low (maximum 0.61 mg L^−1^), resulting in un-ionized ammonia (NH_3_) levels of approximately 0.03 mg L^−1^. This value is well below the chronic toxicity threshold of 0.05–0.1 mg L^−1^ NH_3_ established for freshwater teleosts [23], indicating no risk of ammonia-induced stress. Although alkalinity was maintained within the recommended range of 100–150 mg CaCO_3_ L^−1^, the mean value recorded (193.54 mg L^−1^) is considered beneficial in intensive systems, as it provides greater buffering capacity. This level ensures the efficiency of the nitrification process and prevents abrupt pH fluctuations, offering an additional safety margin against metabolic and environmental variation [45,46].

Regarding nitrogenous compounds, nitrite concentrations remained below 0.05 mg L^−1^ NO_2_–N, well under the critical threshold of 0.5–1.0 mg L^−1^ associated with methemoglobinemia and impaired oxygen transport in freshwater fish [47,48]. Nitrate levels averaged 10.75 mg L^−1^, far below the recommended upper limit of 50 mg L^−1^ for freshwater aquaculture systems, above which chronic exposure may negatively affect growth and health in sensitive species [49]. Total suspended solids (TSS) averaged 21.29 ± 5.67 mg L^−1^, remaining below levels reported to cause adverse effects in fish cultured in recirculating systems [50]. Overall, these water quality parameters confirm that the system maintained suitable conditions for silverside culture throughout the experimental period, preventing the accumulation of nitrogenous metabolites that could otherwise interfere with oxygen consumption dynamics and ammonia excretion rates. This stable environmental background provides confidence that the observed metabolic responses are attributable to the experimental factors (stocking density and body size) rather than to uncontrolled water quality variations.

No previous data have been reported on oxygen consumption or ammonia excretion for *O. bonariensis* under RAS or any other intensive culture system; therefore, this study represents the first record of metabolic rates for this species under recirculating conditions similar to those used in commercial aquaculture. Similarly, no bioengineering metabolic parameters are available for any other species of the family Atherinidae under RAS conditions. This study thus provides the first species-specific metabolic benchmarks for *O. bonariensis*, establishing a reference baseline for RAS system design and sustainable silverside aquaculture intensification.

The static closed-system respirometry approach used in this study eliminated the need for reaeration correction factors (k La), which represent a common source of uncertainty in open-flow or aerated respirometry systems [32,33]. By sealing the tanks completely during each measurement block, all recorded oxygen depletion was attributable exclusively to fish metabolic demand. The 70% air saturation threshold applied (≥6.90 mg O_2_ L^−1^ at the experimental temperature) maintained dissolved oxygen well above the critical partial pressure reported for freshwater teleosts of similar size (~3.5–4.5 mg O_2_ L^−1^) [41], supporting the interpretation that the values obtained reflect routine aerobic metabolism. Although the conventionally recommended threshold for routine metabolic rate measurements is ≥80% air saturation [33], the 70% criterion adopted here is consistent with protocols reported for other cultured teleosts under similar experimental constraints [34], and any potential conservative underestimation of OCR would be uniform across treatments, preserving the validity of intergroup comparisons.

The present study demonstrates that both body size and stocking density significantly influence oxygen consumption rates in *O. bonariensis* cultured under recirculating aquaculture conditions, whereas total ammonia nitrogen (TAN) excretion remains unaffected by these variables. Specifically, smaller individuals exhibited substantially higher mass-specific oxygen consumption compared to larger conspecifics, and fish maintained at lower stocking densities displayed elevated oxygen uptake relative to those reared at higher densities. These findings align with fundamental principles of fish bioenergetics and allometric scaling, wherein metabolic rate per unit mass typically follows a negative power relationship with body size [19,51]. The elevated mass-specific metabolic demands observed in smaller silversides likely reflect their proportionally larger gill surface area relative to body mass, higher cardiac output per gram of tissue, and greater maintenance costs associated with rapid somatic growth during early developmental stages [52,53]. This pattern has been consistently documented across teleost species, including salmonids and cyprinids, where juveniles frequently exhibit oxygen consumption rates 2–4 times higher per gram of body weight than adults [54,55].

In addition to body size, stocking density significantly influenced oxygen consumption rates. Fish maintained at lower densities exhibited higher mass-specific oxygen consumption compared to those held at higher densities. Density-dependent metabolic responses have been widely reported in cultured fish and are often associated with behavioral interactions, space availability, and stress physiology [56,57]. At elevated stocking densities, spatial restriction and intensified social interactions may reduce swimming activity, thereby lowering routine metabolic expenditure [58]. It is hypothesized that prolonged crowding may also elicit chronic stress responses that alter energy allocation patterns, potentially contributing to metabolic suppression as an adaptive mechanism [59,60], a pattern that has been observed in other cultured teleosts. However, none of these behavioral or physiological variables were measured in the present study. The absence of data on swimming activity, plasma cortisol levels, or behavioral interactions represents a recognized limitation, and the observed density-dependent reduction in oxygen consumption should therefore be interpreted cautiously as correlational rather than mechanistic evidence. Conversely, fish maintained at lower densities typically exhibit increased spontaneous activity and exploratory behavior, which in turn elevates routine oxygen consumption [61].

Regarding oxygen consumption, the values obtained in the present study for *O. bonariensis* (ranging from 146.12 to 226.31 mg O_2_ kg^−1^ h^−1^) fall within the ranges reported for other teleost species under similar experimental conditions. Direct interspecific comparison of oxygen consumption rates requires caution, given that metabolic rates are strongly influenced by water temperature, body size, nutritional status, and respirometry methodology. In juvenile gilthead seabream *Sparus aurata* (30–100 g at 21 °C), mean oxygen consumption was 138.9 mg O_2_ kg^−1^ h^−1^ [62]. For rainbow trout (*Oncorhynchus mykiss*), the standard metabolic rate at 10 °C was estimated at 91 mg O_2_ kg^−1^ h^−1^ under normoxic conditions [63], while at 15 °C, values ranged from 96 to 130 mg O_2_ kg^−1^ h^−1^ [64]. In juvenile tench (*Tinca tinca*) (15–19 g at 23 °C), oxygen consumption ranged between 126.80 and 187.35 mg O_2_ kg^−1^ h^−1^ [65]. Juvenile northern pike (*Esox lucius*) (69.2–91.2 g at 20 °C) exhibited mean values ranging from 92.9 to 144.79 mg O_2_ kg^−1^ h^−1^ [66]. For palm fish *Seriolella violacea* juveniles (420 g at 14–18 °C) reared in RAS systems, average values ranged from 66 to 98.4 mg O_2_ kg^−1^ h^−1^ [67]. More recently, in Chinese perch (*Siniperca chuatsi*) (97.84 ± 12.64 g at 23–25 °C), oxygen consumption reached 354.22 ± 26.49 mg O_2_ kg^−1^ h^−1^ [68]. This wide interspecific variability reflects differences in metabolic strategies, ecological niches, and experimental conditions such as temperature, body size, feeding status, and respirometry protocols, underscoring the importance of species-specific metabolic benchmarks for aquaculture system design.

In contrast to oxygen consumption, TAN excretion rates were not significantly affected by stocking density or body size. This apparent decoupling between aerobic metabolic demand and nitrogen excretion suggests that protein catabolism remained relatively stable across treatments, despite differences in routine metabolic expenditure. Ammonia production can serve as a useful indicator of protein metabolism in fish, where protein retention is inversely correlated with ammonia excretion [69]. In teleost fish, ammonia is primarily produced through amino acid deamination associated with protein metabolism and is excreted mainly across the gills via diffusion and active transport mechanisms [70,71]. While oxygen consumption reflects total aerobic energy expenditure—including activity, maintenance, and stress responses—ammonia excretion is more closely linked to dietary protein intake and amino acid turnover [72]. Therefore, when feeding regime and dietary composition are consistent among treatments, nitrogen excretion rates may remain relatively constant even if behavioral activity and oxygen demand vary.

Accordingly, when feeding regime and dietary composition are held constant, nitrogen excretion rates may remain relatively unchanged even under conditions that modify routine metabolic activity. Similar observations have been reported in several teleost species, including *Phoxinus phoxinus*, *T. tinca*, *Acipenser oxyrinchus*, and *Paralichthys californicus*, where body mass had no significant effect on nitrogen excretion [34,73,74,75]. Moreover, previous studies indicate that moderate changes in stocking density do not necessarily alter ammonia excretion unless fish experience severe stress or substantial shifts in protein utilization efficiency [74,76,77]. The absence of density- or size-related effects on TAN excretion in the present study therefore suggests that fish were maintained under metabolic conditions that did not promote increased protein catabolism or nitrogen imbalance.

The mean TAN excretion rates observed across treatments (4.59–10.81 mg kg^−1^ h^−1^) fell within the range reported for several other freshwater teleosts, although notable interspecific variation exists. For instance, in *Notemigonus crysoleucas* (2.5–4.6 g) maintained in recirculating systems, values ranged from 5.2 to 11.2 mg TAN kg^−1^ fish h^−1^ [34]; in juveniles of *Leporinus macrocephalus*, from 2.24 to 38.02 mg TAN kg^−1^ fish h^−1^ [78]; in *T. tinca* juveniles, between 1.95 and 8.80 mg TAN kg^−1^ fish h^−1^ [65]; in *Perca fluviatilis* L. (44.8–336.2 g), from 5.2 to 13.8 mg TAN kg^−1^ fish h^−1^ [79]; and in *Piaractus brachypomus* (46.1–520.0 g), from 79.2 to 177.3 mg TAN kg^−1^ fish h^−1^ [80]. Among marine species reared under aquaculture conditions, *P. californicus* exhibited excretion rates ranging from 3.4 to 4.3 mg TAN kg^−1^ fish h^−1^ [34], while *S. violacea* showed values between 10.70 and 16.40 mg TAN kg^−1^ fish h^−1^ in RAS systems [81]. The wide variability among studies can be attributed to multiple factors, including experimental methods, feed type and composition, feeding rate, stocking density, photoperiod, salinity, dissolved oxygen, fish size, trophic group, and pH, all of which significantly influence fish growth and bioenergetics [29,82,83,84,85,86,87,88].

The apparent nitrogen recovery as TAN (i.e., the proportion of dietary N excreted as ammonia) ranged from 13.2 ± 2.2% (D2S2) to 31.1 ± 10.8% (D1S2) across treatments (Table 2). These values are consistent with those reported for other teleosts fed restricted rations (e.g., 20–35% in *P. californicus* [34]), but lower than those observed under higher feeding rates (40–60%). The relatively low recoveries in the present study likely reflect the moderate feeding level (1% body weight day^−1^), which may have favored nitrogen retention (growth) over catabolic losses. The absence of statistical differences in apparent TAN recovery between treatments (*p* > 0.05) is consistent with the non-significant TAN excretion rates and further suggests that protein catabolism was uniformly moderate across all groups. However, because actual individual feed intake was not measured and fecal N losses, urea excretion, and gill-associated N retention were not quantified, the true nitrogen balance cannot be fully established from these data. The apparent recovery values reported here are therefore conservative estimates of total ammoniotelic N excretion. Future studies should close the full nitrogen balance to refine feed formulations and reduce environmental loading in RAS.

The observed diurnal fluctuations in oxygen consumption and TAN excretion were primarily associated with postprandial metabolic processes, with the most pronounced peaks occurring approximately 2 h after feeding events [89,90]. These fluctuations reflect the specific dynamic action (SDA) response—a transient elevation in metabolic rate driven by the mechanical and biochemical processes of food ingestion, digestion, nutrient absorption, and assimilation [91]. Characteristically, SDA manifests as a rapid increase in oxygen demand following feeding, reaching a peak or plateau before gradually declining as digestive processes progress [88]. The magnitude of these oxygen consumption peaks provides valuable quantitative data that can be leveraged to estimate energy requirements for both fasting catabolism and SDA, thereby enhancing individual-based growth models grounded in bioenergetic budgets [64]. Importantly, while oxygen consumption serves as a direct indicator of total aerobic metabolic expenditure during SDA, the concurrent elevation in ammonia excretion reflects enhanced amino acid deamination associated with protein metabolism. This integrated understanding of oxygen and nitrogen dynamics enables more accurate simulation of catabolic processes that drive ammonia emissions, ultimately supporting refined predictions of both growth performance and nitrogen loading in recirculating aquaculture systems.

Overall, the present study highlights that oxygen consumption in *O. bonariensis* is strongly modulated by both body size and stocking density, whereas TAN excretion remains relatively stable under consistent feeding conditions. From a system design and management perspective, these results suggest that oxygen demand in RAS is more sensitive to population structure and density than ammonia production. These results provide quantitative benchmarks that can support RAS design for *O. bonariensis*. As an illustrative example, for a system maintaining 100 kg of mixed-size fish, the peak oxygen demand (estimated from the maximum observed OCR of 333 mg O_2_ kg^−1^ h^−1^) would be approximately 33.3 g O_2_ h^−1^, which can be used to size aeration equipment given a known oxygen transfer efficiency. Regarding biofilter design, the maximum TAN excretion rate observed (78.63 mg TAN kg^−1^ h^−1^ post-feeding peak) corresponds to a peak TAN load of 7.86 g TAN h^−1^ per 100 kg of fish, which, assuming a standard nitrification rate of 0.1–0.5 g TAN m^−2^ day^−1^ for biofilm media [44], can be used to estimate the required biofilter surface area. However, biofilter sizing should also account for the specific surface area of the media, hydraulic retention time, and the expected nitrification rate, which can range widely (e.g., 0.1–0.5 g TAN m^−2^ day^−1^) depending on temperature and biofilm maturity [44]. These calculations should be validated under site-specific conditions, as biofilter performance is also governed by feed protein content, nitrification efficiency, temperature, alkalinity, and hydraulic retention time, factors not varied in the present study. Biofilter sizing based solely on total biomass, as suggested here, is therefore a starting point rather than a definitive design criterion.

Several limitations of the present study should be acknowledged. First, the experimental design was restricted to two body-size categories and two stocking density levels (2 × 2 factorial), which generated useful baseline benchmarks but did not permit the development of continuous dose–response models or allometric prediction equations across a broader range of sizes and densities. Future studies should employ response-surface or dose-gradient designs spanning a wider range. Second, the broad body-weight ranges used (S1: 48–140 g; S2: >140–250 g) introduced intra-treatment biological variability, which may have reduced statistical sensitivity, particularly for TAN excretion. Third, the limited number of replicate tanks (n = 3 per treatment) constrained statistical power, and the non-significant effects detected for TAN excretion should be interpreted cautiously with respect to potential Type II errors; post hoc power analyses indicated an observed power of approximately 30% for the density effect and 6% for the size effect. Fourth, feed intake was not individually quantified, and fecal nitrogen, urea excretion, and growth nitrogen retention were not measured, precluding a complete nitrogen balance and limiting the interpretation of apparent TAN recovery values. Fifth, behavioral variables (swimming activity, social interactions) and physiological stress indicators (plasma cortisol) were not assessed, preventing mechanistic confirmation of the proposed density-dependent metabolic suppression. Sixth, the experimental duration was limited to 24 h measurement cycles, which does not allow calculation of growth performance indicators such as specific growth rate or feed conversion ratio. These limitations define the scope of the bioengineering parameters reported here and should be addressed in future studies designed to develop predictive models for *O. bonariensis* RAS.

## 5. Conclusions

This study provides the first quantitative measurements of oxygen consumption and ammonia excretion in *O. bonariensis* under RAS conditions. Oxygen consumption was significantly influenced by both body size and stocking density (ranging from 146.12 to 226.31 mg O_2_ kg^−1^ h^−1^), while TAN excretion remained stable across all treatments (4.59–10.81 mg TAN kg^−1^ h^−1^). Both parameters showed pronounced diurnal fluctuations associated with postprandial specific dynamic action. These results provide first-record species-specific benchmarks for oxygen demand (146–226 mg O_2_ kg^−1^ h^−1^ under routine conditions; peak 333 mg O_2_ kg^−1^ h^−1^ postprandially) and ammonia excretion (4.6–10.8 mg TAN kg^−1^ h^−1^ routine; peak 78.6 mg TAN kg^−1^ h^−1^) in *O. bonariensis*, which can serve as starting-point parameters for aeration sizing and biofilter design in RAS under the specific conditions tested and should be validated under site-specific operational conditions, particularly when feeding rates, diet composition, or temperature differ from those reported here.

## Figures and Tables

**Table 1:** Water quality parameters of silverside *Odontesthes bonariensis* reared in a recirculating aquaculture system.

Parameters	D1S1	D1S2	D2S1	D2S2
Temperature °C	16.51 ± 0.06	16.51 ± 0.06	16.52 ± 0.04	16.53 ± 0.05
Dissolved oxygen (mg L^−1^)	9.72 ± 0.07	9.76 ± 0.05	9.73 ± 0.09	9.74 ± 0.06
Oxygen saturation (%)	99.74 ± 0.57	100.18 ± 0.36	99.84 ± 0.77	99.94 ± 0.66
pH	8.96 ± 0.09	8.96 ± 0.09	8.97 ± 0.07	8.98 ± 0.07

Mean ± S.E. No significant differences were detected among treatments for any parameter (*p* > 0.05). D1: low density (3.2 kg m^−3^); D2: high density (6.2 kg m^−3^); S1: small size (48–140 g); S2: large size (>140–250 g).

**Table 2:** Apparent nitrogen balance in *Odontesthes bonariensis* subjected to different stocking densities and fish sizes, based on the feed ration (1% BW day^−1^) and mean TAN excretion rates. Recovery values were calculated under the assumption of complete consumption of the offered feed.

Parameters	D1S1	D1S2	D2S1	D2S2
Dietary N input (mg N g fish^−1^ day^−1^)	0.83	0.83	0.83	0.83
Daily feed intake (g tank^−1^ day^−1^)	16.00 ± 0.07	16.00 ± 0.07	31.00 ± 0.09	31.00 ± 0.09
Apparent TAN recovery (% of dietary N ingested)	27.31 ± 9.01	31.11 ± 10.08	16.03 ± 5.89	13.21 ± 2.23
TAN excreted (mg N tank^−1^ day^−1^)	363.74 ± 119.81	415.12 ± 144.12	414.43 ± 148.84	341.54 ± 57.32

Mean ± S.E. No significant differences were detected among treatments for any parameter (*p* > 0.05). D1: low density (3.2 kg m^−3^); D2: high density (6.2 kg m^−3^); S1: small size (48–140 g); S2: large size (>140–250 g).

## Data Availability

Data will be made available upon request.
